# Highlights from the Tenth European Breast Cancer Conference (EBCC10), Amsterdam, 9–11 March 2016

**DOI:** 10.3332/ecancer.2016.644

**Published:** 2016-06-03

**Authors:** Joana Ribeiro, Maria João Cardoso

**Affiliations:** 1 Breast Unit, Champalimaud Clinical Center/Champalimaud Foundation, Lisbon 1400-038, Portugal

**Keywords:** breast cancer, early, advanced, diagnostic local treatment, systemic treatment

## Abstract

The Tenth European Breast Cancer Conference, EBCC 10, was held in Amsterdam in March 2016 with a total of 3061 participants from 95 different countries spread over five continents.

The EBCC council is a joint venture of ESO, EORTC Breast Cancer Group, EUSOMA, and Europa Donna. The Scientific Programme for EBCC-10 tried to bring to the stage all the active participants in the diagnostic and treatment of breast cancer along with patients themselves.

The need to achieve ‘patient’s access to high quality treatment’ through breast units that have been accredited through a European certification process was the basis of the EBCC 10 manifesto.

The congress scientific programme allowed participants to review the most up-to-date knowledge in the breast cancer field presented by experts having in mind its application to every day practice.

The purpose of this summary is to describe to the readers the results of the late-breaking and best abstracts presented at EBCC10.

## EBCC 10 manifesto

The EBCC 10 manifesto with the subsequent call-to-action was presented by Fatima Cardoso, the Congress President. Dr Cardoso stressed that the 2016 deadline for all patients in European Union countries to access specialists, multidisciplinary breast cancer units, or centres, will be missed by most countries despite numerous resolutions and declarations issued since the year 2000 that have called for universal specialist services. This means that many women and some men still do not receive optimal breast cancer care in Europe.

The EBCC10’s call to action was directed towards policymakers and politicians. They together with healthcare professionals and patient advocates were to promote, in public and professional settings, the evidence that breast units staffed with specialist multidisciplinary teams deliver superior care and quality of life (QoL) to women and men with breast cancer. This also in order to acknowledge and strengthen the evidence that treatment in multidisciplinary units leads to overall cost savings as well as higher QoL.

## Screening

Prof Nehmat Houssami from the Sydney School of Public Health presented the results of the ASTOUND study—Adjunct Screening with Tomosynthesis or Ultrasound (US) in Women with Mammography—Negative Dense Breasts [1]. The ASTOUND is a prospective study of adjunct screening which can be used to compare incremental breast cancer (BC) detection by tomosynthesis and ultrasound in women with mammography-negative dense breasts, having BC detection rate as end point. The study was based on Italian population recruited from five Italian centres with dedicated breast imaging facilities. Women aged ≥38 years with dense breasts were included. A total of 3231 of them were analysed. Twenty four additional BC were detected: 12 on tomosynthesis and US, only 1 on tomosynthesis, only 11 on US. Tomosynthesis and US detected more BC in women with negative 2D-mammogram and dense breasts. US detected more BCs than tomosynthesis however >50% of additional BCs in 2D-negative cohort detected by integrating tomosynthesis (3D) in same screening procedure. The authors concluded that tomosynthesis acquisition reconstructs high-quality 2D image, and as a consequence has the potential to avoid double-acquisition. Tomosynthesis-based screening mammography can be the future for women with dense breasts, maybe sparing the necessity of adjunct screening.

## Locoregional treatment

In the last decade surgery and radiotherapy (RT) have been moving towards less aggressive/more conservative approaches given the low recurrence rates and high overall survival (OS) based upon the results derived from the latest overviews on breast cancer outcomes [2]. Charlotte Coles on behalf of the trial co-authors presented the results of the IMPORT LOW [3]. The trial is a randomised multicentre phase III trial testing partial breast RT, i.e. using intensity modulated RT in women with low risk early stage breast cancer (EBC). In these woman it is found that late complications of RT are the dominant hazard rather than local recurrence (LR). Patients with small, low risk tumours were randomised (1:1:1) to 40 Gy/15 F to whole breast (control), 36 Gy/15 F to whole breast, and 40 Gy/15 F to partial breast (test 1); or 40 Gy/15 F to partial breast (test 2). The primary endpoint was local tumour control in the ipsilateral breast. A total of 2018 patients were recruited from May 2007 to September 2010 from 30 UK RT centres (675 control, 674 test 1, 669 test 2). Median follow-up is 68.3 (IQR 60.3–73.4) months. The five-year rate of LR was 1.1% (95% CI 0.5, 2.3), 0.2% (95% CI 0.02, 1.2), and 0.5% (95% CI 0.2−1.4) in the control, test 1, and test 2 groups respectively. Absolute treatment differences in LR with control compared with test 1 is−0.83% (95% CI−1.04, 0.18) and−0.69% (−0.99, 0.44) compared with test 2. For each of the test groups non-inferiority, assessed against the pre-specified 2.5% threshold was demonstrated. As conclusion authors stated that at five years, partial breast RT was shown to be non-inferior to whole breast RT in women with low risk EBC. LR rates were very low in all treatment groups and moderate and marked normal tissue events were also low across all groups.

In line with the results from the ACOSOG Z11 and AMAROS trial the OTOASOR—optimal treatment of the axilla after positive sentinel lymph node biopsy in primary invasive breast cancer patients (surgery versus radiotherapy)—a randomised clinical trial with ten years follow-up was presented by Dr Savolt from Budapest [4]. The trial compares completion axillary lymph node dissection (cALND) to axillary nodal irradiation (ANI) in patients with sentinel lymph node (SLN)-positive primary invasive breast cancer. Patients with primary invasive breast cancer (clinically lymph node negative and less than or equal to 3 cm in size) were randomised before surgery for cALND (arm A-standard treatment) or ANI (arm B-investigational treatment).

There were 474 patients analysed in the study. The clinical and tumuor characteristics were similar between 244 patients randomised to cALND and 230 randomised to SLNB plus ANI. The primary endpoint of the study was axillary recurrence and secondary endpoints were OS, breast cancer specific survival, disease-free survival (DFS), distant disease-free survival (dDFS). Mean length of follow-up was found to be 107 months. Axillary recurrence (primary endpoint) was 1.6% versus 1.7% (p = NS). The 9.1 year OS was 84.9% versus 91.2%; ten-year DFS was 79.9% with cALND and 85.6% with SLND plus ANI. The authors concluded that at ten years follow-up the results from OTOASOR trial suggest that ANI without cALND does not increase the risk of axillary failure in SLN+ patients. These results came in the way of the previous studies and again stressed that axillary disease is usually minimal in this early breast cancer setting and that surgical dissection when compared with axillary radiotherapy does not bring any higher local control. Not only that, it can also have a more detrimental effect on arm function and cause lymphoedema.

Immediate breast reconstruction after mastectomy is becoming nowadays more frequent and should be offered to every woman when there are no major contraindications. The rise in skin sparing mastectomies and the popularisation of Acellular Dermal Matrices (ADM) have progressively replaced the two-stage breast reconstruction (expander–implant in two interventions). The one stage breast reconstruction is with a permanent implant and more frequently with an ADM coverage.

However the evidence justifying the use of ADMs in implant-based breast reconstruction (IBBR) is limited. The study presented by Dr Dikmans on behalf of all co-authors is a Dutch prospective multicentre non randomised trial aiming at comparing the outcomes of direct IBBR augmented with an ADM with those of two-stage IBBR [5]. The trial was conducted at eight hospitals in the Netherlands. Patients who intended to undergo skin-sparing mastectomy and immediate IBBR were randomised to one of the two procedures. The authors at EBCC 10 presented the effect of the procedure on the occurrence of adverse outcomes. Between 14 April 2013, and 29 May 2015, 140 patients were enrolled in the study. Among them, 59 patients (91 breasts) in the one stage IBBR group and 59 (87 breasts) in the two-stage IBBR group were included for analysis. The overall medical complication rates (38.5% versus 10.3%, OR = 6.28, p = 0.001), the medical re-operation rates (32.6% versus 9.6%, OR = 3.96, p = 0.009) and the implant explantation rates (27.0% versus 2.4%, OR = 15.17, p = 0.001) were significantly higher in the one-stage group. This remained the case after controlling for multiple confounding factors (p < 0.001). The authors concluded that immediate one-stage ADM-assisted IBBR was associated with a significantly higher rate of postoperative complications compared with two-stage IBBR. These results indicate that immediate one-stage ADM assisted IBBR should be considered very carefully. Those are interesting results that must be dealt carefully because of the limited size of the sample. The results of the Multicentre Canadian Acellular Dermal Matrix Trial (MCCAT) will probably help us to better understand the outcomes of these two procedures [6].

## Systemic treatment

A meta-analysis of two large randomised phase III clinical trials that compared adjuvant chemotherapy (CT) given every two weeks (dose-dense (dd) regimen) or every three weeks (the standard interval) was presented at EBCC10 by Dr Lambertini [7]. This meta-analysis looked at the efficacy of ddCT in the subgroup of premenopausal BC patients, and its impact on the risk of developing treatment-induced amenorrhea (TIA). The two studies: MIG1 and GIM2 enrolled 1549 pre-menopausal high-risk (lymph node negative and positive) breast cancer patients [8, 9]. There findings suggest superiority of ddCT as compared to standard interval regimens in premenopausal patients at higher risk of relapse with a significant improvement in OS after ten years by nearly a third (29%). This benefit was irrespective of hormone receptor status: ten-year OS was improved by 22% in patients with hormone receptor positive tumours (HR+ve), and by 35% in those with hormone receptor negative (HR-ve) tumours.

Regarding TIA two aspects should be highlighted. First ddCT was not associated with an increased risk of TIA. Second its occurrence did not affect survival, although there was a non-statistically significant trend towards improved survival for women with hormone receptor positive tumours who developed TIA.

In the HER-2 setting new data from the EPHOS-B trial was reported [10]. This trial was designed to evaluate the effect of 10 to 12 days of preoperative anti-HER-2 therapy (lapatinib and trastuzumab alone or in combination) on biomarkers of proliferation (Ki67). The study enrolled 257 women with operable newly diagnosed HER-2-positive (HER-2+ve) breast cancer. In the first part, 130 patients were randomised to no perioperative treatment (control), trastuzumab only (6 mg/kg on days one and eight pre-surgery) or lapatinib only (1500 mg/day). However, as evidence emerged from other trials on the efficacy of dual anti-HER-2 therapy, the trial was amended to enroll additional 127 patients to control, trastuzumab monotherapy, or dual anti-HER-2 therapy with lapatinib (1000 mg/day) and trastuzumab.

The results from the second part of the trial reported at this conference showed that 11% of patients that received lapatinib plus trastuzumab had pathological complete response (pCR) and an additional 17% had minimal residual disease (MRD). In contrast, for women who only received trastuzumab, 1% had pCR and 2% had MRD. No patients had either pCR or MRD in the control group.

The results of this important trial confirm previous initial suggestions that probably there are patients who can be treated with dual anti-HER-2 therapy alone, i.e. without chemotherapy. This study proposes a simple way to identify those patients very early on, which could help spare them unnecessary chemotherapy. However, it is necessary to confirm if these early responses translate into better or equally long-term survival in larger studies.

## Genomic test/biomarkers

### Plan B trial

Reassuring data for patients with HR+ve and low recurrence score (RS ≤ 11) by the Oncotype DX cases were presented by the West German Study Group (WSG) at this conference. The phase III Plan B trial [11] enrolled 3198 patients, median age of 56 years; with node negative or node positive (1–3 nodes) HR+ve and HER-2 negative EBC. These patients had a clinically-determined intermediate or high risk of recurrences.

For 348 patients (15.3%) with an RS ≤ 11, adjuvant chemotherapy was omitted and endocrine therapy alone was performed. With a median follow-up of 55 months the authors reported a five-year DFS of 94% for this group of patients, showing that it is possible to use a multigene test to identify patients with clinically-determined intermediate or high risk of recurrent EBC, i.e. those who can be spared chemotherapy.

Analysis of other prognostic factors, such as nodal status, tumour size, grade and Ki67 showed that the 21-gene RS was a better independent predictor of disease recurrence than the clinical factors alone or Ki67. This very important trial adds to the evidence from the low-risk arm of the TAILORx study, and to a wealth of other retrospective studies, supporting the use of genomic testing to help accurately select EBC patients who can safely be spared CT.

### MINDACT trial

A study looking at the association between multifocality (MF) and genomic risk (using Mammaprint) in the subgroup of clinical low risk patients (as defined by the Adjuvant!Online based cut-off) in the MINDACT protocol was discussed [12]. The authors identified 3326 clinical low risk patients from which 238 had MF. A significant association between genomic risk and MF was found—the percentage of genomic high risk increased from 17.3% for unifocal to 22.7% for MF tumours—this corresponds to an absolute increase by 5.4% and a relative increase of 31%. Apart from an absolute increase in the frequency of lobular tumours by 10% (17.9% versus 7.8% by central and 21.8% versus 10.8% by local pathology), the authors did not observe any differences in tumour and in patient’s baseline characteristics for MF versus unifocal tumours. In multivariable regression analysis multifocal disease remained significantly associated with a high-risk 70-GS result (OR 1.64, 95% CI 1.03–2.08, p = 0.035).

### USO 01062 trial

A study exploring the molecular landscape of clinical high-risk EBC using a comprehensive biomarker analysis of patient’s samples from the Phase III—United States Oncology trial 01062—was presented at Amsterdam [13]. In this adjuvant trial 2611 patients were randomised to receive standard anthracycline-taxane containing chemotherapy regimen with or without capecitabine.

Researchers used DNA and RNA extracted from 1539 breast cancer samples and performed multiplex gene expression, copy number, and qPCR mutation assays. Once again five year DFS in PAM50 subtypes was as predicted. Of note there was a trend for better DFS with the addition of capecitabine in the basal subtype. Profiling of immunologic genes allowed the identification of a distinct set of immune genes that were associated with DFS in HR+ve and TN BC population.

## Male breast cancer

Male breast cancer (Male BC) biology was in focus at this conference. Carolien van Deurzen reported results from a sub-study of the International Male Breast Cancer Programme looking at the relationship between the pathology of different types of Male BC and their prognosis [14]. The authors examined 1203 tumour samples from Male BC patients—the largest series of this disease ever collected linked to outcome data — and found that the development of fibrotic connective tissue and the density of tumour infiltrating lymphocytes were strongly associated with outcomes in Male BC. On the other hand, tumour grade, a commonly used prognostic measure in female breast cancer was not related with prognosis. Like in previous analysis [15] researchers confirmed that the great majority of Male BC are luminal being the other subtypes (HER-2+ve and triple negative) extremely rare. Given the rarity of this disease the authors encouraged not only worldwide collaboration for the development of trials in this setting but also enrolment of Male BC patients in general breast cancer trials.

## Metastatic breast cancer

A report from the chair of the conference, Fatima Cardoso, about the global status of metastatic breast cancer was presented at this year’s EBCC [16]. The report mainly focused on the scientific advances/medical interventions achieved in this setting, but it also more importanly identified the major needs of these patients were related to psychosocial support and QoL issues. In fact the data showed that in 2013 eight out of ten women felt that QoL was the biggest area in need of improvement. Other aspects associated with communication issues, societal attitudes, caregivers services ,and the cost burden of this disease were also analysed.

## Conclusion

The EBCC 2016 took place in an environment that can be characterised as stimulating discussion and practical approaches to breast cancer diagnosis and treatment. Key topics were reviewed by experts and various clinical case discussions (Multidisciplinary Teams in the Real World) in the early and advanced settings were undertaken. This clearly was an enriching experience to one and all. Important presentations in usually overlooked themes such as Male Breast cancer were highlighted. Above all this year’s conference was firmly based on a holistic vision of breast cancer, and it was successful in bringing together scientists, clinicians, patient representatives, and healthcare professionals of diverse profiles to tackle the key issues that patients face throughout the continuum of a breast cancer journey.
